# CD160 mediated NK cell activation shapes host defense against acute *Trypanosoma cruzi* infection in the skin

**DOI:** 10.3389/fimmu.2026.1872132

**Published:** 2026-09-22

**Authors:** Jessica Barton, Cari Lehmann, Ulricke Richardt, Dániel Cadar, Beate Volkmer, Rüdiger Greinert, Thomas Jacobs, Rosa Isela Gálvez

**Affiliations:** 1 Protozoa Immunology, Bernhard Nocht Institute for Tropical Medicine, Hamburg, Germany; 2 Virus Metagenomics and Evolution, Bernhard Nocht Institute for Tropical Medicine, Hamburg, Germany; 3 Arbovirology and Entomology, Bernhard Nocht Institute for Tropical Medicine, Hamburg, Germany; 4 Skin Cancer Center, Division of Molecular Cell Biology, Elbe Klinikum Buxtehude, Elbe Klinikum Stade-Buxtehude, Buxtehude, Germany

**Keywords:** CD160 and HVEM, Chagas, natural killer cells, skin immunity, *Trypanosoma cruzi*

## Abstract

**Introduction:**

*Trypanosoma cruzi*, the causative agent of Chagas disease, enters the host through the skin. While natural killer (NK) cell antimicrobial activity against *T. cruzi*-infected keratinocytes in the epidermis has previously been shown, NK cell interactions with dermal fibroblasts, a primary target of parasite replication at the skin entry site, remain poorly understood.

**Methods:**

Bulk RNA-sequencing of mock- and *T. cruzi*-infected human dermal fibroblasts (HDF) and the BJ fibroblast cell line was performed to identify infection-induced ligand changes. HVEM-CD160 binding and NK cell effector functions were assessed in vitro using fibroblast co-cultures with or without CD160 receptor blockade. *In vivo*, RAG^-/-^ and RAG^-/-^CD160^-/-^ mice were infected intradermally with *T. cruzi*; NK cell degranulation and cytokine production were analyzed by flow cytometry, and parasite burden in skin and muscle was quantified by qPCR.

**Results:**

RNA-sequencing revealed upregulation of herpesvirus entry mediator (HVEM, TNFRSF14) in infected human dermal fibroblasts, confirmed at the protein level. HVEM was functionally engaged by CD160, and CD160 blockade reduced NK cell degranulation and secretion of cytotoxic mediators in fibroblast co-cultures. CD160 deficiency impaired NK cell activation *in vivo*; NK cell degranulation in the skin showed a trend toward inverse correlation with local parasite burden, and early skin parasite load predicted subsequent dissemination to muscle. HVEM was robustly upregulated in murine skin during acute infection, mirroring the human fibroblast data.

**Discussion:**

These findings establish the HVEM-CD160 axis as a key regulator of NK cell effector function in the skin during acute *T. cruzi* infection, contributing to local parasite control. This conserved human-mouse regulatory response may offer broader insight into NK cell-mediated defense against pathogens that invade through the skin.

## Introduction

*Trypanosoma cruzi* (*T. cruzi*), the causative agent of Chagas disease, is an obligate intracellular protozoan parasite that primarily enters the host through the skin following a triatomine insect bite (1–3). Dermal fibroblasts are among the first cell types infected, serving as early reservoirs for parasite replication before systemic dissemination (4–6). Early immune responses at the site of entry are therefore critical for limiting parasite burden and shaping the course of infection. Despite its central role, the innate defense mechanisms in the skin during *T. cruzi* infection remain poorly understood. Natural Killer (NK) cells are cytotoxic lymphocytes that act as first responders in peripheral tissues, including the skin, where they detect and eliminate infected or stressed cells (7, 8). NK cell effector functions include cytolytic granule release containing perforin, granzyme A, and granzyme B, as well as cytokine secretion such as IFN-γ, which contributes to macrophage activation and enhanced antigen presentation (9). Granule-mediated cytotoxicity is initiated upon target recognition, leading to perforin pore formation, granzyme entry, and apoptosis of infected cells (10–12). NK cell activation is tightly controlled by a balance between inhibitory and activating receptors, integrating signals from MHC-I molecules and stress-induced ligands on target cells (13, 14).

The skin epidermis primarily provides a physical barrier function, while the dermis is vastly traversed by blood vessels and lymphatics, allowing recruitment of both innate and adaptive immune cells that facilitate its participation in cell mediated immunity, with NK cells acting in both the epidermis and dermis to detect and eliminate infected cells (15, 16). Skin structure and immune function change over the course of life, with age dependent differences in barrier integrity and immune responsiveness. As the primary site of inoculation for vector-borne pathogens, including *T. cruzi*, we and others follow the hypothesis that the skin likely shapes infection outcome and disease course, and represents an overlooked target for therapeutic interventions (17–19).

In peripheral tissues like the skin, NK cells are strategically positioned to provide early control of pathogens such as *T. cruzi*, potentially limiting systemic dissemination to organs including skeletal muscle and heart. Conventional circulating NK cells can be recruited to infected skin and differentiate into long-lived tissue-resident NK cells with memory-like properties exhibiting enhanced IFN-γ, perforin, and granzyme responses upon subsequent challenge (20). These observations suggest that early NK cell activity at the site of pathogen entry may play a pivotal role in controlling parasite burden and restricting systemic spread. Building on our previous studies in keratinocytes (21), we aimed to investigate NK cell interactions with dermal fibroblasts, a major cellular target of *T. cruzi* in the skin.

Expression of HVEM on BJ fibroblasts increased over the course of *T. cruzi* infection, suggesting a potential role as a ligand for NK cell activation via CD160. By focusing on HVEM-CD160 interactions, we sought to elucidate how dermal fibroblasts contribute to early NK cell-mediated defense and parasite control in the skin. CD160 is a receptor of the immunoglobulin superfamily expressed on NK cells, subsets of T cells, intraepithelial lymphocytes, and endothelial cells (22–24). On NK cells, CD160 engagement consistently promotes cytotoxicity and IFN-γ production (25–27). The receptor exists as a GPI-anchored isoform or, on activated NK cells, as a transmembrane variant (28, 29). GPI-anchored CD160 lacks intrinsic signaling domains and transduces activation signals via association with co-receptors such as CD2 in lipid rafts (30). CD160 interacts with classical and non-classical MHC-I molecules and with HVEM with high affinity (31). While HVEM also binds BTLA and LIGHT, NK cell activation via CD160 predominates, particularly in infection contexts (32).

Although CD160 mediated NK cell activation is well established in systemic viral infections, its role during skin infections, particularly *T. cruzi* invasion, remains largely unknown. It is unclear whether CD160 engagement influences NK cell degranulation and IFN-γ production in the skin, and how this may impact early parasite control and dissemination to other tissues such as muscle.

We hypothesized that early NK cell responses in the skin, particularly in the dermis where fibroblasts serve as primary parasite targets, are critical for preventing systemic immunosuppression and limiting parasite spread and represent a potential target for modulating early parasite control. Furthermore, the HVEM-CD160 axis likely plays a central role in regulating NK cell effector functions in the dermis during acute *T. cruzi* infection. This study aimed to investigate: (i) the expression of HVEM in murine skin and human dermal fibroblasts upon infection, (ii) the effects of CD160 blockade on NK cell responses to infected human dermal fibroblasts *in vitro*, (iii) the functional consequences of CD160 deficiency on NK cell activation *in vivo*, and (iv) the capacity of skin-resident NK cells to degranulate (CD107a) and secrete IFN-γ in response to infection. Together, these experiments define the mechanistic contribution of the HVEM-CD160 interaction to early immune defense against *T. cruzi* at the skin entry site.

## Results

### HVEM is upregulated in human dermal fibroblasts upon *T. cruzi* infection

We performed bulk RNA-sequencing of mock- and *T. cruzi* infected primary human dermal fibroblasts (HDF) and the fibroblast cell line BJ to evaluate the suitability of BJ fibroblasts for an *in vitro* model of a dermal *T. cruzi* infection. In addition, the gene signatures after *T. cruzi* infection should provide information on possible mechanisms for the modulation of NK cell activity. Transcriptome analysis revealed a significant increase in expression of the HVEM encoding gene TNFRSF14 following *T. cruzi* infection of both HDF and BJ fibroblasts cell line at 48 hours post-infection (hpi). In the BJ cell line, TNFRSF14 expression increased from BJ mock ≈ 8.05 Reads Per Kilobase of transcript per Million mapped reads (RPKM) to BJ infected ≈ 10.94 RPKM (~1.36-fold) at 48 hpi. In primary HDFs, which showed variable baseline expression, all five donors exhibited an infection-associated increase, with fold-changes ranging from 1.18 to 1.85. When all samples (BJ and HDFs) were analyzed together (combined), HVEM expression was significantly higher in infected cells compared with paired mock samples (paired t-test, p = 0.0027; Wilcoxon matched-pairs signed-rank test, p = 0.0312) (**Figure 1A**). Importantly, this effect remained significant when considering HDFs alone (paired t-test, p = 0.0106); a Wilcoxon signed-rank test on this subset showed a consistent increase in all five donors (p = 0.0625, the minimum possible exact p-value at n = 5). Based on these results, further analyses were performed using BJ fibroblasts exclusively. HVEM protein expression on BJ fibroblasts was assessed by flow cytometry between 24 and 72 hpi. Technical replicates (n = 3) were averaged per experiment (n = 3 independent experiments per condition/time point). Data were analyzed by two-way mixed-effects model with Sidak’s multiple comparisons test. No significant difference between mock and infected cells was observed at 24 hpi (p = 0.466). HVEM upregulation became significant at 48 hpi (p = 0.023) and further increased by 72 hpi (p = 0.0056) **Figure 1B**), indicating that HVEM induction develops progressively over the course of infection.

**Figure 1:**
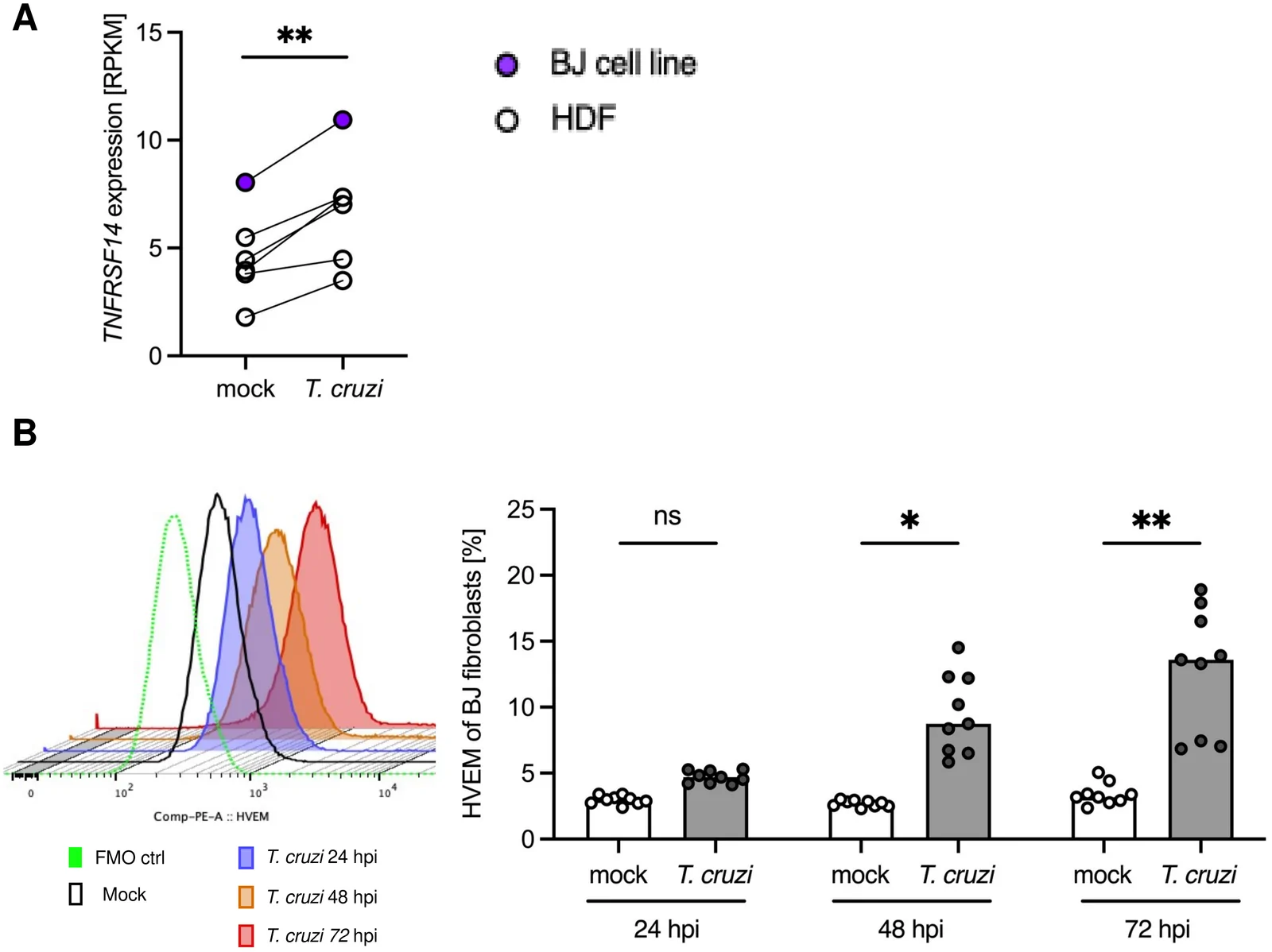
HVEM is upregulated in human dermal fibroblasts in response to *T. cruzi* infection. **(A)** Transcriptome analysis shows increased HVEM expression in BJ fibroblasts and HDFs in response to *T. cruzi* infection. RNA sequencing was performed on mock and *T. cruzi* infected fibroblasts (5 HDF donors and BJ cell line fibroblasts) at 48 hpi (MOI = 3). RPKM values depict HVEM (TNFRSF14 gene) expression. Normality was assessed using the Shapiro–Wilk test, and data were analyzed with a two-tailed paired t-test (p = 0.0027 for combined analysis, p = 0.0106 for HDFs alone); Wilcoxon matched-pairs signed-rank tests confirmed these results (p = 0.0312 and p = 0.0625, respectively). **(B)** Flow cytometry analysis of surface HVEM expression over the course of infection (24, 48, 72 ah). Left: Representative flow cytometry histograms. Right: Data represent three independent biological experiments (n = 3), with three technical replicates averaged within each experiment prior to analysis. Statistical differences were calculated using a two-way mixed-effects model (REML) with time and infection as fixed effects and experiment as a random effect, followed by Sidak’s multiple comparisons test to compare infected and mock-treated samples at each time point. Significance: ns (24 hpi, p = 0.466), *p = 0.023 (48 hpi), **p = 0.0056 (72 hpi).

### Infection-induced HVEM is functionally engaged by CD160

To assess whether HVEM upregulated on infected fibroblasts is capable of engaging the NK cell stimulatory receptor CD160, binding of recombinant human CD160 (hrCD160) to BJ fibroblasts was examined at 48 hpi (**Figures 2A, B**). Binding of hrCD160 was significantly increased on *T. cruzi* infected fibroblasts compared with mock controls (n = 8; two-tailed Mann-Whitney test, ***p = 0.0002). These results indicate that HVEM upregulated during infection is accessible and capable of functionally interacting with CD160 in *trans*, providing a mechanistic link to the subsequent NK cell activation observed in co-culture experiments.

**Figure 2:**
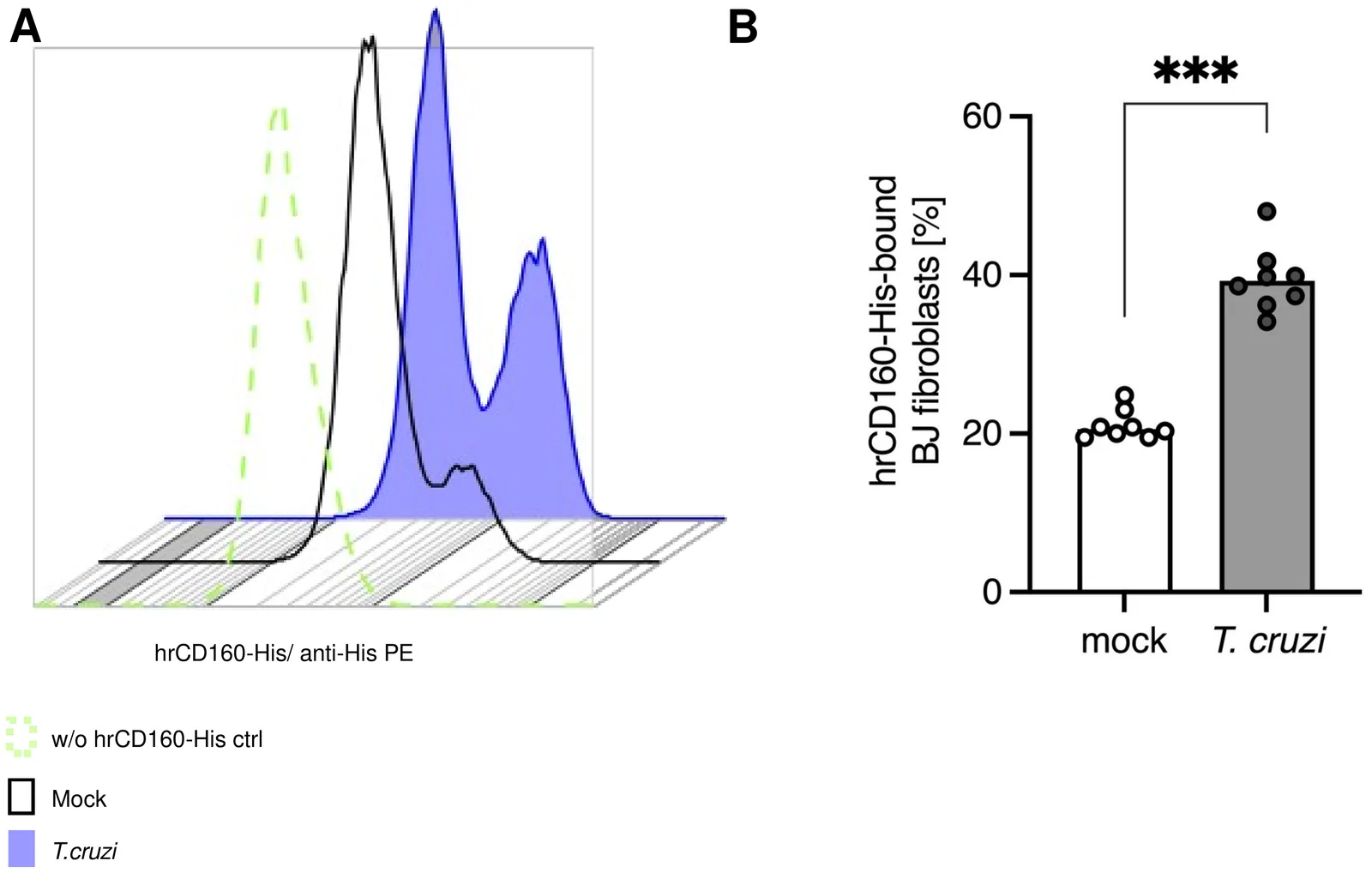
Increased binding of CD160 to human dermal fibroblasts in response to *T. cruzi* infection. Human recombinant CD160 (hrCD160) exhibits an increased binding to infected fibroblasts, demonstrating functional interaction of the HVEM-CD160 axis during infection. **(A)** Representative flow cytometry histograms showing hrCD160 binding to infected vs. mock fibroblasts at 48 hpi. **(B)** Frequency of hrCD160-His–bound BJ fibroblasts 48 hpi (MOI = 3), Bars indicate medians (n = 8). Statistical significance was calculated using a two-tailed Mann-Whitney test: ***p = 0.0002.

### CD160 blockade dampens the NK effector functions *in vitro*

To assess the role of CD160 in NK cell activation, NK cells were co-cultured with either mock-treated *or T. cruzi* infected BJ fibroblasts with or without prior CD160 blockade. **Supplementary Figure 1A** provides an overview of the *in vitro* experimental workflow used. Receptor blockade efficiency was confirmed by reduced CD160 surface staining on NK cells compared with untreated controls (**Figure 3A**). CD160 blockade reduced NK cell degranulation (CD107a^+^) in co-cultures with mock-treated fibroblasts (paired t-test, p = 0.0014) and, to a greater extent, in co-cultures with infected fibroblasts, decreasing the frequency of CD107a^+^ NK cells by >60% (paired t-test, p < 0.0001) (**Figure 3B**). Because a larger fraction of NK cells degranulated in response to infected fibroblasts at baseline, the absolute number of NK cells affected by CD160 blockade was greater in infected co-cultures. CD160 blockade also significantly reduced secretion of the cytotoxic mediators granzyme A (mock: p = 0.0043; infected: p = 0.0078), granzyme B (mock: p = 0.0078; infected: p = 0.0078), perforin (mock: p = 0.0017; infected: p = 0.0033), granulysin (mock: p = 0.0078; infected: p = 0.0001), sFasL (mock: p = 0.0036; infected: p = 0.0078), and IFN-γ (mock: p = 0.0078; infected: p = 0.0004), in co-cultures with both infected and mock-treated fibroblasts (**Figure 4**). The magnitude of this reduction was greater in co-cultures with infected fibroblasts, reflecting their higher baseline NK cell activation. Together, these results indicate that CD160 blockade dampens NK cell effector functions toward BJ fibroblasts irrespective of infection status, with a greater number of NK cells affected in the context of *T. cruzi* infection, consistent with the increased baseline activation induced by infected fibroblasts.

**Figure 3:**
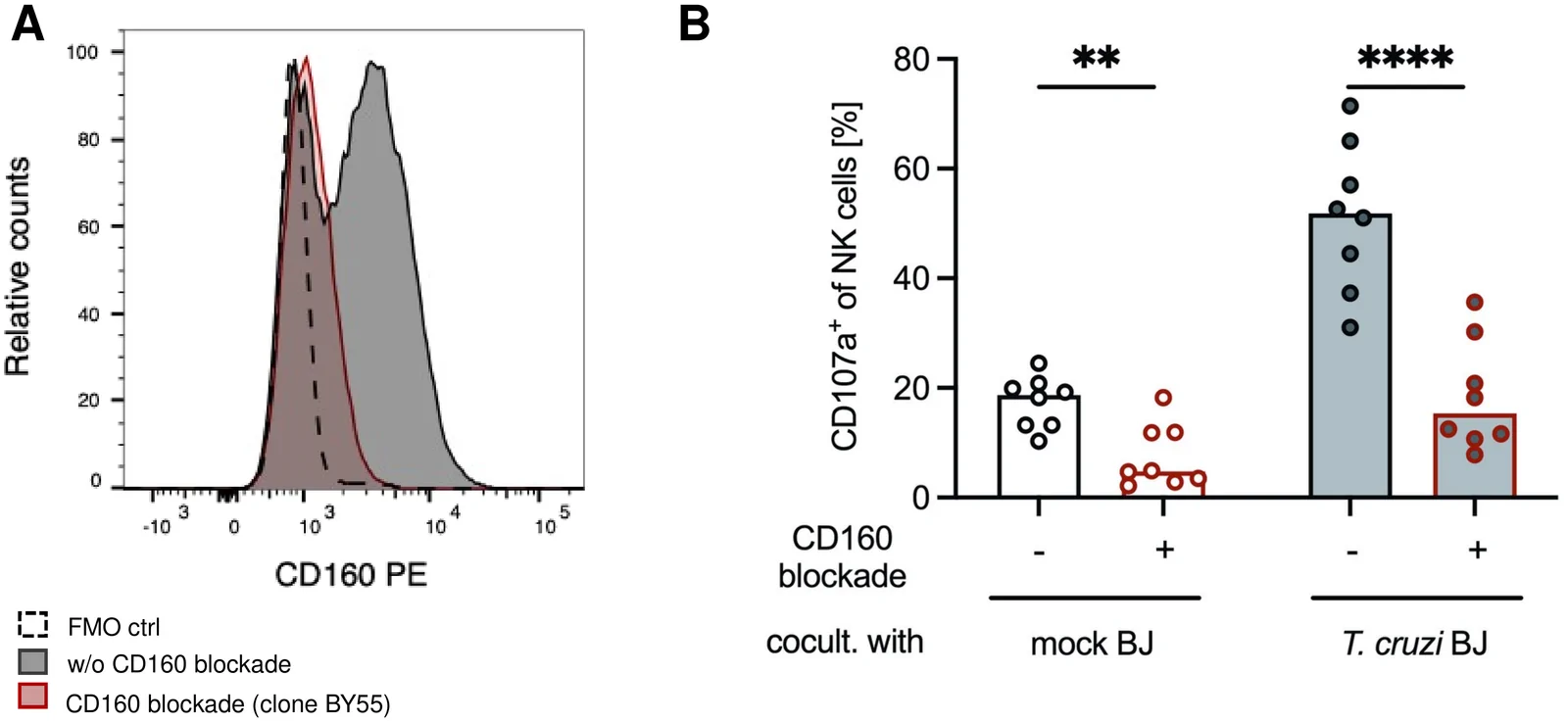
CD160 blockade reduces NK cell degranulation in response to *T. cruzi* infected human dermal fibroblasts. **(A)** Flow cytometry histogram showing reduced staining of CD160 on NK cells (CD3^−^CD56^+^) after pre-treatment with a CD160-blocking antibody prior to co-culture with fibroblasts. Representative histogram demonstrates effective receptor blockade. **(B)** NK cell degranulation measured as CD107a surface staining after 24 ah co-culture with mock or *T. cruzi* infected BJ fibroblasts (MOI = 3; E:T ratio = 1:10; n = 8), ± CD160 blockade. Means ± SD are shown as bars. Statistical significance was calculated using paired t-tests: mock BJ ± blockade, **p = 0.0014; *T. cruzi* BJ ± blockade, ****p < 0.0001.

**Figure 4:**
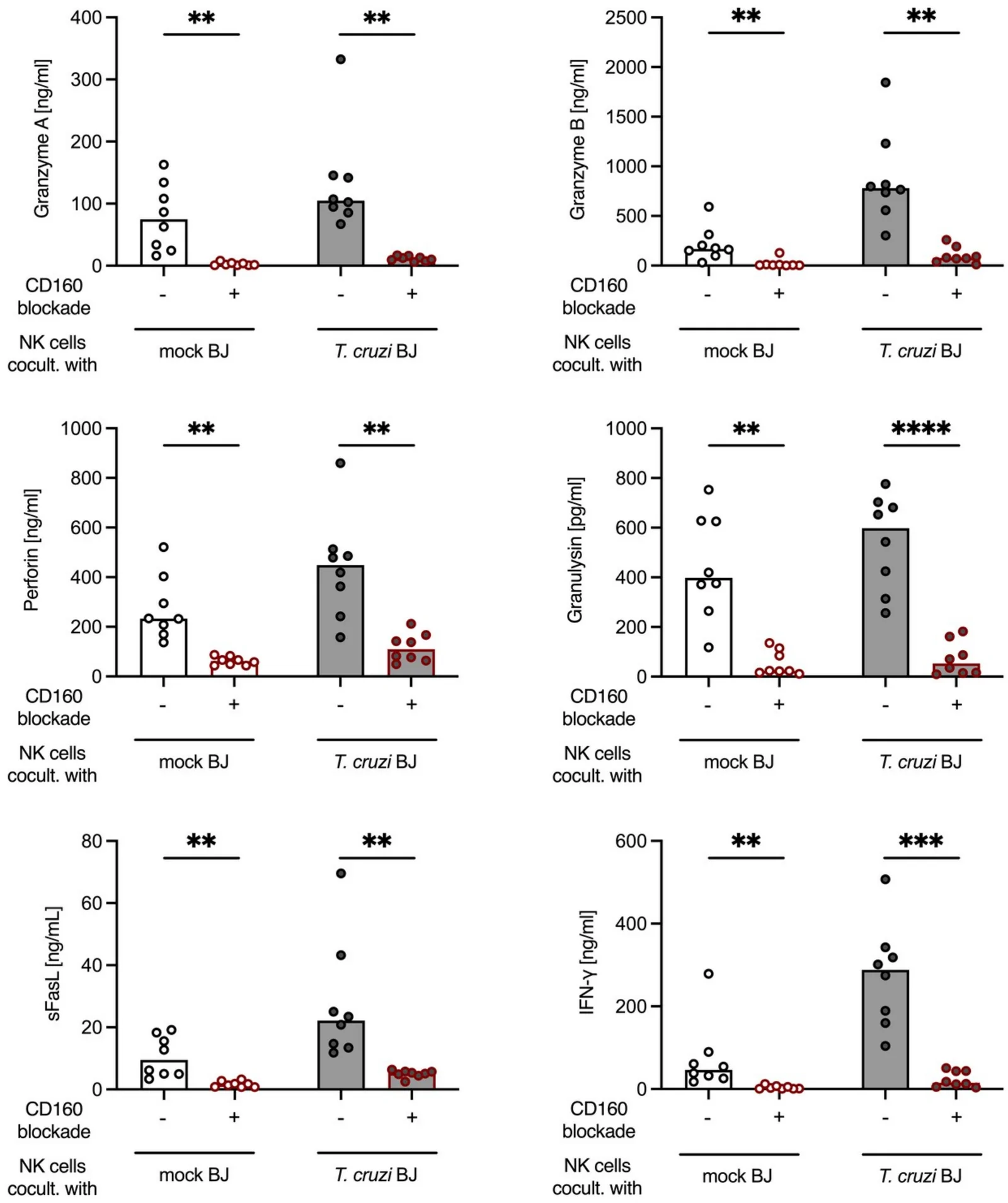
CD160 blockade reduces NK cell effector cytokines and cytolytic mediators in response to *T. cruzi* infected fibroblasts. Concentrations of granzyme A, granzyme B, perforin, granulysin, sFasL, and IFN-γ in supernatants after 24 ah co-culture with mock- or *T. cruzi* infected BJ fibroblasts (MOI = 3; E:T ratio = 1:2.5; n = 8), ± CD160 blockade. Medians are shown. Normality of paired differences was assessed for each comparison individually (Shapiro-Wilk test); paired t-tests were used where normality was not rejected, and Wilcoxon matched-pairs signed-rank tests were used otherwise (see Methods). **p ≤ 0.01; ***p ≤ 0.001; ****p ≤ 0.0001.

### HVEM is selectively induced in murine skin *in vivo*

To extend our findings from human dermal fibroblasts to an *in vivo* model, we analyzed HVEM expression in the skin and muscle of RAG^-/-^ mice at day 7 post-infection (dpi) (experimental design: **Supplementary Figure 2A**). HVEM mRNA was significantly increased in the skin of infected mice compared with naive controls (median fold-change 15.3 vs. 1.3; Mann-Whitney, n = 3 naive, n = 6 infected, p = 0.048, **Figure 5A**), whereas muscle tissue from the same mice showed substantially lower HVEM expression (**Supplementary Figure 2B**).

**Figure 5:**
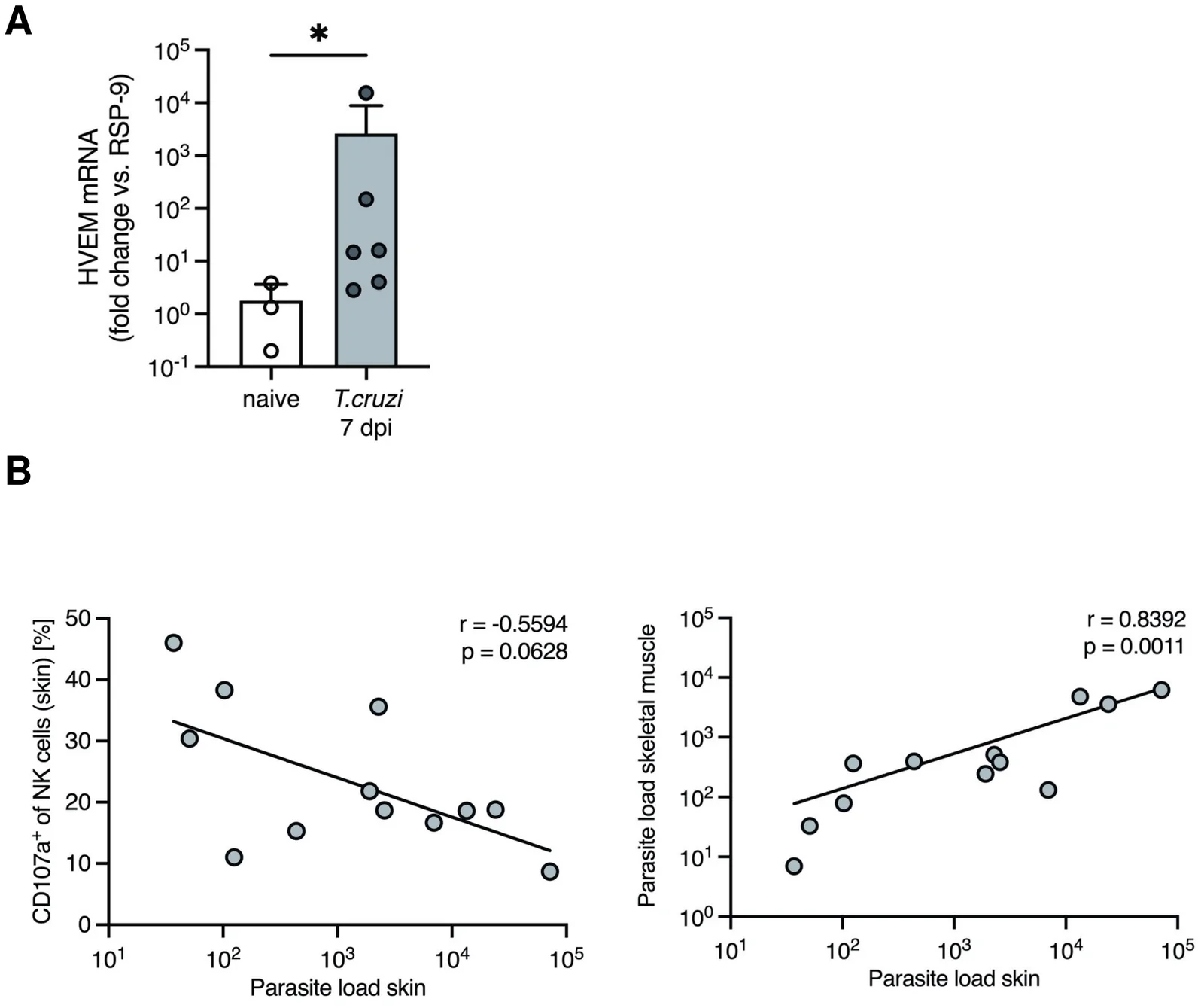
HVEM (TNFRSF14) expression and NK cell activity in the skin during acute *T. cruzi* infection of RAG^-/-^ mice. **(A)** Fold change of HVEM (TNFRSF14) mRNA in skin relative to naive controls, normalized to RSP-9, at 7 dpi in RAG^-/-^ mice. Each point represents an individual mouse (n = 6 infected, n = 3 naive), pooled from two independent experiments. HVEM expression was markedly higher in skin of infected mice compared to naive controls. Mann-Whitney U test confirmed a significant increase in HVEM expression in skin (p = 0.048). Data are presented as median with interquartile range. **(B)** Correlation analyses of NK cell activity and parasite burden during acute infection (n = 12 infected mice, pooled from two independent experiments). Left: Spearman correlation between skin NK cell CD107a degranulation and skin parasite load (r = -0.5594, p = 0.0628). Right: Spearman correlation between skin parasite load and muscle parasite load (r = 0.8392, p = 0.0011). *p= 0.048.

### Skin NK cell degranulation correlates with local and systemic parasite burden

Parasite load in the skin and muscle of acutely infected RAG^-/-^ mice was quantified at 7 dpi (**Figure 5B**). Skin NK cell degranulation (CD107a) showed an inverse correlation with skin parasite burden but this was not significant (Spearman correlation analysis, r = -0.5594, p = 0.0628). Skin parasite burden positively correlated with muscle parasite burden and this was significant (r = 0.8392, p = 0.0011). Skin NK cell degranulation also showed a non-significant trend toward an inverse correlation with muscle parasite burden (r = -0.5804, p = 0.0521**;** **Supplementary Figure 2C**).

### CD160 deficiency impairs NK activation during acute infection *in vivo*

To assess the role of CD160 *in vivo*, NK cell activation in response to acute *T. cruzi* infection was compared between RAG^-/-^ and RAG^-/-^ CD160^-/-^ mice at 7 dpi (experimental design in **Supplementary Figure 3A**, gating strategy in **Supplementary Figure 4A**).

Because the skin represents the primary site of parasite entry but is highly vascularized, it cannot be excluded that circulating NK cells contribute to the cell population detected in skin samples at the time of tissue harvest. Therefore, NK cell activation was additionally assessed in peripheral blood as a marker of systemic NK cell activation and recruitment, as we do in human studies and measured by degranulation (CD107a) as well as by IFN-γ production. While NK cell degranulation showed only a non-significant reduction in RAG^-/-^ CD160^-/-^ mice compared with RAG^-/-^ controls (p = 0.2315), IFN-γ production was significantly decreased (p = 0.0150), indicating impaired NK cell effector function in the absence of CD160 (**Figure 6A**).

**Figure 6:**
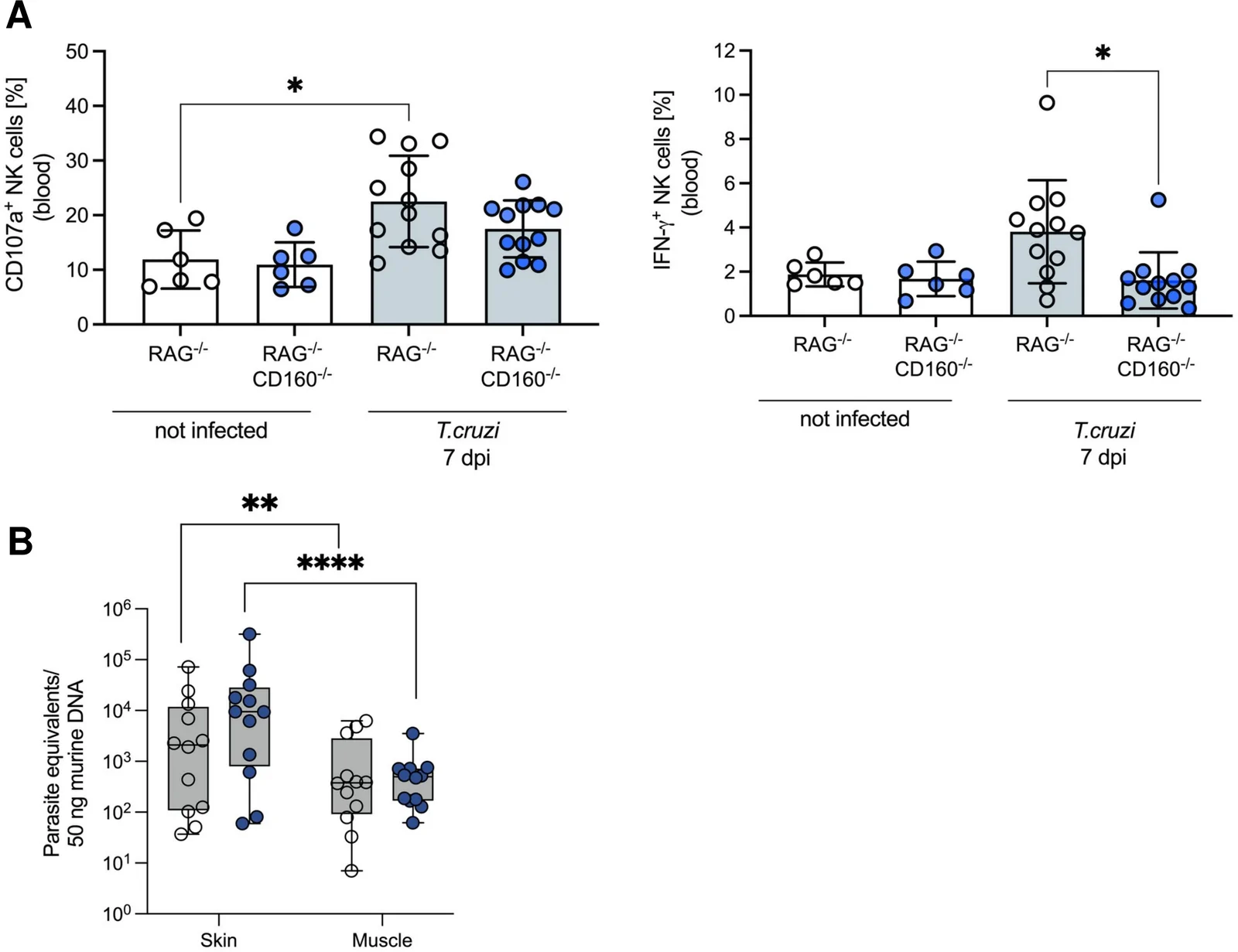
CD160 deficiency reduces NK cell activation in response to acute *T. cruzi* infection in RAG^-/-^ mice. **(A)** Left: Quantification of CD107a^+^ NK cells (degranulation) in peripheral blood of naive (n = 6) and *T. cruzi* infected (7 dpi; n = 12) RAG^-/-^ and RAG^-/-^ CD160^-/-^ mice. Each point represents an individual mouse. Statistical comparisons across all four groups were performed using the Kruskal-Wallis test followed by Dunn’s multiple comparisons test. Degranulation increased significantly upon infection in RAG^-/-^ mice (p = 0.0165); RAG^-/-^ vs. RAG^-/-^ CD160^-/-^ (infected) showed only a trend toward reduction (p = 0.2315, ns). Right: Quantification of IFN-γ NK cells in peripheral blood in the same groups, showing a significant reduction in cytokine-producing NK cells in RAG^-/-^ CD160^-/-^ mice compared to RAG^-/-^ controls (p = 0.0150). **(B)** Parasite burden in skin and muscle tissues at day 7 post-infection in RAG^-/-^ and RAG^-/-^ CD160^-/-^ mice. Parasite equivalents per 50 ng genomic DNA are shown on a log10 scale; bars indicate medians. Statistical analysis was performed on log10-transformed values using a two-way mixed-effects model (REML; tissue as repeated measures, genotype as between-subjects factor) with Bonferroni-corrected multiple comparisons. Skin vs. muscle: RAG^-/-^, **p = 0.0091; RAG^-/-^ CD160^-/-^, ****p < 0.0001. RAG^-/-^ vs. RAG^-/-^ CD160^-/-^: skin, p = 0.115 (ns); muscle, p = 0.888 (ns).

Parasite burden was significantly higher in skin than in muscle for both genotypes (RAG^-/-^: **p = 0.0091; RAG^-/-^ CD160^-/-^: ****p < 0.0001). Direct comparison between genotypes revealed a tendency toward higher parasite loads in the skin of RAG^-/-^ CD160^-/-^ mice compared with RAG^-/-^ controls, although this difference did not reach statistical significance (p = 0.115); parasite levels in muscle were likewise comparable between genotypes (p = 0.888) (**Figure 6B**). Absolute numbers of activated NK cells were quantified in skin and muscle across all genotypes and infection states (**Table 1**). CD107a^+^ NK cell counts were broadly similar between skin and muscle across groups (median range: 4.8×10^4^–2.0×10^5^ in skin vs. 2.6×10^4^–8.6×10^4^ in muscle). In contrast, IFN-γ NK cell counts were consistently higher in skin than in muscle by approximately two orders of magnitude across all groups (median range: 3.8×10^4^–1.3×10^5^ in skin vs. 8.1×10²–2.1×10³ in muscle), indicating that IFN-γ production by NK cells is strongly concentrated at the cutaneous site of infection, whereas degranulation capacity is more evenly distributed between tissues. Kruskal-Wallis testing did not reveal significant differences in absolute NK cell counts across genotype and infection status within either tissue for either marker (**Supplementary Figures 5A, B**).

**Table 1:** Absolute NK cell effector responses in skin and skeletal muscle.

	Skin	Muscle	Skin	Muscle
	CD107a^+^	CD107a^+^	IFN-γ^+^	IFN-γ^+^
RAG⁻/⁻ naive	4.8×10^4^	2.6×10^4^	3.8×10^4^	1.6×10^3^
RAG⁻/⁻ infected	6.6×10^4^	5.2×10^4^	1.3×10^5^	2.1×10^3^
RAG⁻/⁻CD160⁻/⁻ naive	2.0×10^5^	6.0×10^4^	5.7×10^4^	8.1×10^2^
RAG⁻/⁻CD160⁻/⁻ infected	7.0×10^4^	8.6×10^4^	8.4×10^4^	1.0×10^3^

Analysis of CD11b and CD27 expression (overview: **Supplementary Figures 6A, Kruskal**-Wallis test across all four groups per subset, followed by Dunn’s multiple comparisons test; statistical comparisons: **Figures 7A–D**) revealed no significant difference in NK cell subset distribution between RAG^-/-^ and RAG^-/-^ CD160^-/-^ mice at baseline. Following *T. cruzi* infection, RAG^-/-^ CD160^-/-^ mice showed a significant reduction in the CD11b^−^CD27^−^ (p = 0.0271; **Figure 7A**), CD11b^−^CD27^+^ (p < 0.0001; **Figure 7B**), and CD11b^+^CD27^+^ (p = 0.0181; **Figure 7C**) subsets compared with RAG^-/-^ controls, accompanied by a significant increase in the terminally mature CD11b^+^CD27^−^ subset (82.2% vs. 69.9%, p < 0.0001; **Figure 7D**).

**Figure 7:**
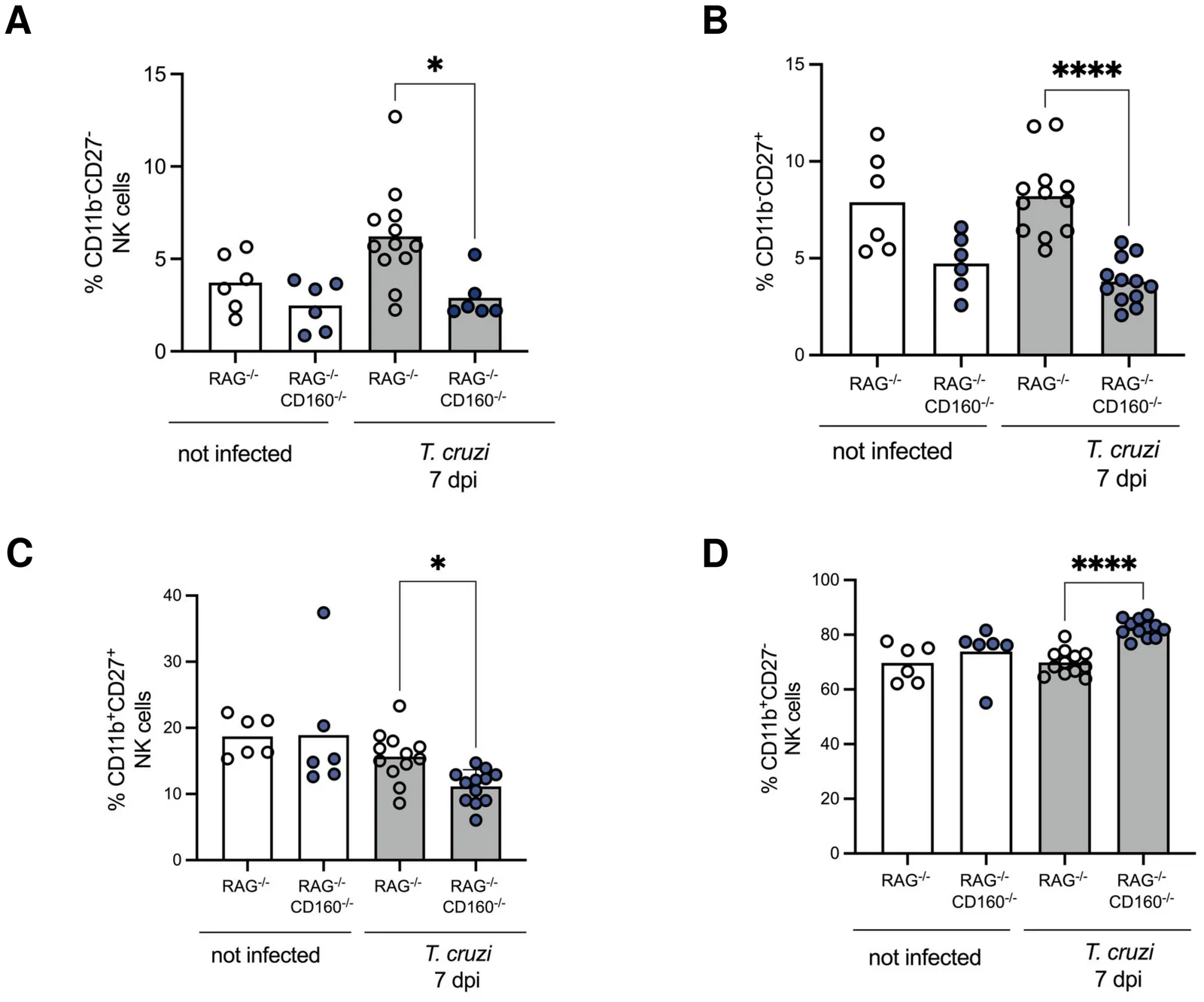
CD160 deficiency alters the NK cell maturation landscape. Skin NK cells (CD3^−^NK1.1^+^) from RAG^-/-^ and RAG^-/-^ CD160^-/-^ mice were analyzed for CD11b and CD27 expression and 7 dpi *T. cruzi* infection. **(A)** Frequency of CD11b^−^CD27^−^ NK cells. **(B)** Frequency of CD11b^−^CD27^+^ NK cells. **(C)** Frequency of CD11b^+^CD27^+^ NK cells. **(D)** Frequency of CD11b^+^CD27^−^ (terminally mature) NK cells. Each point represents an individual mouse (not infected: n = 6 per genotype; infected: n = 12 per genotype). Bars indicate medians. Statistical comparisons across all four groups were performed using the Kruskal-Wallis test followed by Dunn’s multiple comparisons test. *p < 0.05; ****p < 0.0001.

### HVEM-CD160 signaling is conserved across human and murine skin

Together, the human and murine data support a consistent model. In human dermal fibroblasts, *T. cruzi* infection increased HVEM (TNFRSF14) expression by 1.2 to 1.9-fold at the transcript level (p = 0.0027 combined, p = 0.0106 for HDFs alone), with a corresponding progressive increase at the protein level over 72 hpi (p = 0.023 at 48 hpi, p = 0.0056 at 72 hpi), enabling infected cells increasingly to engage CD160 (p = 0.0002; **Figures 1**, **2**). This axis was functionally required for NK cell effector activity *in vitro*: CD160 blockade reduced degranulation (p = 0.0014 mock, p < 0.0001 infected) and IFN-γ secretion (p = 0.0078 mock, p = 0.0004 infected), an effect most pronounced in co-cultures with infected fibroblasts (**Figures 3**, **4**). *In vivo*, HVEM mRNA was similarly induced in murine skin during acute infection (p = 0.048) while remaining low in muscle, mirroring the tissue-restricted induction observed in human fibroblasts. Consistent with a functional role for CD160 in this tissue-specific response, RAG^-/-^ CD160^-/-^ mice showed impaired NK cell IFN-γ production in peripheral blood (p = 0.0150) and a broader, statistically significant shift across all four NK cell maturation subsets specifically during infection (p ≤ 0.0271 for all subsets; **Figures 6A**, **7A–D**). Together, these human and murine findings support a unified model in which infection-induced HVEM on skin stromal cells engages CD160 on NK cells as an early, tissue-restricted checkpoint that shapes both the magnitude and the differentiation trajectory of local NK cell responses to *T. cruzi*.

## Discussion

Increasing evidence indicates that tissue-specific signals directly influence innate and adaptive immune responses, yet the mechanisms governing early immune activation within barrier tissues remain incompletely understood. Stromal and epithelial cells are increasingly recognized as central organizers of local immunity, forming specialized niches that regulate immune cell recruitment, differentiation, and activation. Dermal fibroblasts, in particular, actively shape the skin immune landscape through mutual interactions with resident immune cells, indicating that stromal cells are dynamic immunological participants rather than passive structural elements (33, 34).

In the present study, we investigated the HVEM-CD160 axis as the main driver of early NK cell responses at the site of *T. cruzi* inoculation in the skin. Bulk RNA sequencing revealed a strong upregulation of HVEM (TNFRSF14) in both primary human dermal fibroblasts and the foreskin-derived BJ fibroblast line, establishing the cell line as a mechanistically tractable and translationally relevant *in vitro* model. At the protein level, HVEM expression on BJ fibroblasts increased progressively over 72 hpi, becoming statistically significant from 48 hpi onward (p = 0.023) and further increasing by 72 hpi (p = 0.0056), confirming our transcriptional data and demonstrating that *T. cruzi* infection increased ligand availability. Functionally, we could show that recombinant human CD160 exhibit a significantly increased binding to infected fibroblasts, indicating that HVEM upregulated during infection is accessible in *trans* and thus fully competent to engage CD160 expressed on NK cell.

CD160 blockade in co-culture experiments strongly reduced NK cell degranulation (CD107a^+^), cytotoxic mediator release (granzyme A, granzyme B, perforin, granulysin, sFasL), and IFN-γ production. Although CD160 blockade also partially affected NK responses to mock-treated fibroblasts, the effect was strongly increased in infected co-cultures, highlighting that infection amplifies HVEM-CD160 dependent NK cell activation and subsequent effector functions. These findings support the notion that HVEM-CD160 interactions modulate NK cell function in response to pathogen-induced fibroblast activation.

Following this mechanistic link, *in vivo*, an acute *T. cruzi* infection induced marked upregulation of HVEM in murine skin of mice, whereas muscle tissue exhibited comparatively low expression. In peripheral blood, NK cell IFN-γ production was significantly impaired in RAG^-/-^ CD160^-/-^ mice (p = 0.0150), with only a trend toward reduced degranulation (p = 0.2315). Furthermore, skin parasite load positively correlated with muscle parasite burden (r = 0.8392, p = 0.0011), suggesting that early NK cell responses at the entry site may limit systemic dissemination, although a direct comparison of skin parasite burden between genotypes did not reach statistical significance (p = 0.115). This confirms a functional contribution of this pathway to early innate defense despite relatively low baseline CD160 expression on murine NK cells when compared to human NK cells. Interestingly, CD160 deficiency also shifted NK cell maturation toward a terminal CD11b^+^CD27^−^ phenotype (p < 0.0001), reducing the proportion of intermediate cytokine producing subsets, with significant reductions also observed in the CD11b^−^CD27^−^, CD11b^−^CD27^+^, and CD11b^+^CD27^+^ subsets (p ≤ 0.0271 for all). This altered subset distribution likely contributes to diminished IFN-γ secretion and emphasizes that CD160 not only regulates NK cell activation magnitude but may also influence differentiation and effector potential during acute infection.

Several broader implications emerge from this work. First, early NK cell activation via HVEM-CD160 may establish an inflammatory milieu that primes subsequent adaptive immune responses, potentially influencing long-term parasite control and tissue-specific immunity. Second, our data underscore the importance of receptor-ligand specificity in tissue contexts: HVEM induction was largely restricted to skin, highlighting the critical role of localized stromal signaling in modulating NK function. Third, CD160 likely operates within a complex network of co-stimulatory and co-inhibitory pathways, integrating additional signals to fine-tune NK cell responses; future studies dissecting this network are essential to fully understand NK cell regulation in skin and other peripheral tissues.

## Conclusion

In conclusion, our findings establish the HVEM-CD160 axis as a tissue-specific checkpoint that couples fibroblast sensing of *T. cruzi* infection to rapid, localized NK cell activation at the skin entry site, thereby contributing to early parasite control. This work advances current understanding of skin-resident innate immunity and Chagas disease pathogenesis by identifying dermal fibroblasts as active sentinels that instruct NK cell effector function and differentiation during acute infection, rather than serving as passive structural bystanders. Future studies extending this axis into the chronic phase of infection, and characterizing human tissue-resident NK populations *in situ*, will be important to determine whether therapeutic modulation of HVEM-CD160 signaling could be leveraged to enhance early parasite control in Chagas disease.

## Limitations of the study

Several limitations from our work should be acknowledged. First, our *in vivo* analyses were restricted to the acute phase in a mouse model, as RAG^-/-^ mice do not survive *T. cruzi* infection. Consequently, we were unable to address chronic disease stages, which are central to the development of Chagas disease. Our data therefore primarily reflect early immune responses in the skin and do not capture the complexity of chronic tissue pathology or adaptive immune regulation. In addition, murine NK cells have been reported to express lower levels of CD160 than human NK cells (26, 27). It is therefore plausible that the CD160 pathway contributes more prominently to human NK cell regulation than our murine model can reveal, and that our *in vivo* data underestimate the magnitude of the effect observed in the *in vitro* co-cultures. Second, our *in vitro* experiments relied on a reductionist co-culture system consisting of human fibroblasts and NK cells that were not autologous. This approach does not recapitulate the structural and cellular complexity of intact human skin, which includes keratinocytes, endothelial cells, tissue-resident immune populations, and extracellular matrix components. The absence of additional cytokine-producing cells and three-dimensional tissue architecture may influence NK cell activation thresholds and receptor-ligand dynamics. More physiologically relevant organotypic skin models or 3D organoid systems will be required to fully understand spatial and microenvironmental regulation of NK cells. Third, NK cells were analyzed only at day 7 post-infection and were broadly defined as NK1.1^+^CD45^+^CD3^−^ lymphocytes. This strategy does not distinguish between conventional circulating NK cells and tissue-resident NK or ILC1 populations. It therefore remains unclear whether the observed responses derive from recruited NK cells, locally differentiating populations, or long-lived tissue-resident subsets. Future studies incorporating refined phenotyping and transcriptional profiling will be essential to resolve NK cell heterogeneity, persistence, and potential memory-like features beyond the acute phase.

## Resource availability

Lead contact

Further information and requests for resources and reagents should be directed to and will be fulfilled by the lead contact, Rosa Isela Gálvez (rosa.galvez@bnitm.de).

Materials availability

All materials generated in this study will be available upon request and execution of a material transfer agreement (MTA) with the Lead Contact.

Data and code availability

RNA sequencing data generated in this study have been deposited in the NCBI Gene Expression Omnibus under accession GSE [#######]. All other data supporting the findings of this study are included within the article and Supplemental Information. No custom computational code was generated.

Human subjects

Peripheral blood was collected from healthy adult donors (n = 8; age 24–39 years; 4 females, 4 males) at the Bernhard Nocht Institute for Tropical Medicine (BNITM), Hamburg, Germany. Human dermal fibroblasts (HDFs) were isolated from skin biopsies of six healthy adult donors (age 52–61 years; 3 females, 3 males) at Elbe Klinikum Buxtehude, Germany. Written informed consent was obtained for all participants. The project was approved by the ethics committee of the Elbe Kliniken Stade-Buxtehude.

Experimental mice model

C57BL/6J RAG^-/-^ and RAG^-/-^ CD160^-/-^ double knockout mice were maintained under specific pathogen-free conditions at BNITM. Both sexes aged 8–12 weeks were used. All experiments were approved by the City of Hamburg Office for Consumer Protection (permit numbers 56/13, 32/15, 52/17) and complied with German animal welfare regulations.

*In vitro T. cruzi* Infection of fibroblasts

BJ fibroblasts were seeded at 4 × 10^4^ cells/cm² 24 ah prior to infection. Fibroblasts were infected with *T. cruzi* Brazil strain trypomastigotes at a multiplicity of infection (MOI) of 3:1. After 24 ah, cultures were washed with DPBS and maintained in fresh medium. Cells were harvested at 24, 48, and 72 ah post-infection (hpi) for RNA extraction, flow cytometry and receptor binding assays.

RNA sequencing

Total RNA from mock and *T. cruzi* infected BJ fibroblasts and HDFs (48 hpi, MOI = 3) was extracted using the RNeasy Mini Kit (Qiagen). RNA integrity was confirmed using the Agilent Bioanalyzer (RIN >8). Libraries were prepared with the QIAseq Stranded mRNA Select Kit and sequenced on an Illumina NextSeq 550 (75 bp paired-end). Reads were mapped to GRCh38, quantified as RPKM, and differential gene expression analyzed for TNFRSF14 (HVEM) using GraphPad Prism 9.

Flow cytometry

Fibroblasts and NK cells were stained in FACS buffer (DPBS + 2% FCS + 2 mM EDTA) for surface markers and viability. For intracellular staining, fixation/permeabilization kits (Foxp3/Transcription Factor Staining Set) were used. Flow cytometry data were acquired on BD LSRFortessa or Cytek Aurora and analyzed in FlowJo v10.8.

Recombinant CD160 binding assay

Mock- and *T. cruzi* infected fibroblasts (48 hpi) were incubated with His-tagged human recombinant CD160 (hrCD160-His) for 1 ah. Bound protein was detected with PE-conjugated anti-His secondary antibody. Gating was based on fibroblasts stained with secondary antibody alone.

NK cell isolation and co-culture

PBMCs were isolated from human blood using Ficoll-Paque in SepMate tubes. NK cells were purified via MojoSort negative selection (>90% CD3^−^CD56^+^) and rested overnight in RPMI-1640 + 10% FCS + 5 ng/mL IL-15. NK cells were pre-incubated with anti-CD160 (clone BY55, 20 μg/mL) or isotype control for 30 min at 4 °C, washed, and blockade efficiency was confirmed by residual CD160 surface staining (FMO control). NK cells were co-cultured with mock- or *T. cruzi* infected fibroblasts (MOI = 3) for 24 ah at an effector-to-target (E:T) ratio of 1:2.5 - 1:10. Anti-CD107a antibody was added at the start of co-culture; degranulation was assessed by surface CD107a staining after 24 ah. Supernatants were analyzed using the LEGENDplex Human CD8/NK Panel and LEGENDplex Data Analysis Software.

*In vivo* infection and tissue processing

Mice were infected intradermally at the tail base with 2 × 10³ *T. cruzi* trypomastigotes in 30 μL PBS. Skin and muscle were collected at indicated time points. Muscles were weighed, and divided for PCR (25 mg) and RNA (25 mg) analysis. Tissues were minced and digested in 5 mL DMEM containing 0.2% Collagenase II and 0.05% Dispase II and incubated for 20–30 min at 37 °C with shaking. Homogenates were mechanically dissociated with a pipette, and enzymatic digestion was stopped with 5 mL FCS. Samples were filtered through 70 μm strainers, centrifuged at 1600 rpm for 5 min at 4 °C, resuspended in PBS containing 1% FCS, and counted. Cell suspensions were aliquoted for FACS analysis (5 × 10^6^ cells per sample). Skin Tissue hair over the tail base was shaved, and two 2 × 2 cm sections were collected for flow cytometry and DNA/RNA analysis. Skin samples were minced and digested in Dermis Dissociation Buffer containing 1× Collagenase P and 1× DNase I. Digestion was performed in two 30-min intervals at 37 °C with pipetting every 30 min, for a total incubation time ≤90 min. Homogenates were filtered through 40 μm strainers, washed with DMEM containing 10% FCS, centrifuged at 400 × g for 5 min at 4 °C, resuspended in PBS containing 1% FCS, counted, and prepared for NK cell flow staining. Parasite load was determined by qPCR for *T. cruzi* satellite DNA, normalized to GAPDH as described before (35).

Quantification and statistical analysis

Statistical analyses were performed using GraphPad Prism 11.0.2. Normality was assessed using the Shapiro-Wilk test (and, where sample size permitted, D'Agostino-Pearson, Anderson-Darling, and Kolmogorov-Smirnov tests). Parametric tests were used where normality was not rejected; non-parametric alternatives were used otherwise. For datasets with small sample sizes where standard normality tests could not yield informative results ("N too small"), this is noted in the corresponding figure legend. P < 0.05 was considered significant.

**Figure 1A**: RPKM values were compared using a two-tailed paired t-test (combined BJ+HDF analysis and HDF-only analysis). Given the small sample size (n = 6 and n = 5, respectively), Wilcoxon matched-pairs signed-rank tests were additionally performed as robustness checks, confirming both results.

**Figure 1B**: Technical replicates (n = 3 per experiment) were averaged within each of three independent experiments prior to analysis (final n = 3 experiments per condition and time point). A two-way mixed-effects model (REML) was applied, with time and infection as fixed effects and experiment as a random effect, followed by Sidak's multiple comparisons test at each time point.

**Figure 2B**: Normality could not be reliably assessed at n = 8 per group; a two-tailed Mann-Whitney test was used.

**Figures 3B**, **4A**: Normality of paired differences was assessed for each comparison individually. Paired t-tests were used where normality was not rejected; Wilcoxon matched-pairs signed-rank tests were used otherwise (see individual figure legends for test assignment per comparison).

**Figure 5A**: Group sizes were unequal (n = 3 naive, n = 6 infected) and normality could not be reliably assessed; a two-tailed Mann-Whitney test was used.

**Figure 5B**: Correlations were assessed using two-tailed Spearman correlation tests.

**Figure 6A**: Statistical comparisons across all four groups (genotype × infection status) were performed using the Kruskal-Wallis test, followed by Dunn's multiple comparisons test.

**Figure 6B**: Parasite burden data (skin and muscle) were log10-transformed prior to analysis to stabilize variance given their wide dynamic range. A two-way mixed-effects model (REML) was used, with tissue as a repeated-measures factor and genotype as a between-subjects factor, followed by Bonferroni-corrected multiple comparisons for the pre-specified comparisons of interest (skin vs. muscle within each genotype; genotype comparison within each tissue).

**Figures 7A–D**: Due to small sample sizes in the naive groups, normal distribution could not be reliably assessed. Statistical comparisons across all four groups (genotype × infection status) were performed using the Kruskal-Wallis test, followed by Dunn's multiple comparisons test.

**Supplementary Figures S5A–B**: Statistical comparisons across all four groups within each panel were performed using the Kruskal-Wallis test; no *post-hoc* testing was performed given the non-significant omnibus result.

## Data Availability

The original contributions presented in the study are publicly available. This data can be found here: NCBI SRA, accession number PRJNA1526664.

## Supplementary material

Supplementary Figure 1 Graphical abstract of *in vitro* experimental workflow. **(A)** Schematic overview of the *in vitro* experimental design. Fibroblasts were infected *in vitro*. In parallel, peripheral blood mononuclear cells (PBMCs) were isolated, followed by natural killer (NK) cell enrichment. NK cells were subsequently co-cultured with infected fibroblasts. Functional modulation was performed using antibody-mediated receptor blocking. Experimental readouts included flow cytometry analysis to assess cellular phenotypes and activation status, as well as cytokine assays to quantify secreted immune mediators.

Supplementary Figure 2 HVEM expression and NK cell responses in RAG^-/-^ mice during infection. **(A)** Schematic overview of the *in vivo* experimental design using RAG^-/-^ mice, (n = 6, n=12 mice per group). HVEM expression was assessed in infected tissues. Parasite burden in skin and skeletal muscle was quantified by PCR. In parallel, NK cell activity was evaluated by measuring degranulation (CD107a expression) and IFN-γ production. **(B)** Relative HVEM mRNA expression levels (fold change) in skin and skeletal muscle following infection. Data demonstrate a markedly higher induction of HVEM expression in skin compared to muscle tissue. **(C)** Correlation analysis between NK cell degranulation in skin (CD107a^+^ NK cells) and parasite load in skeletal muscle. A negative correlation was observed (r = -0.5804, p = 0.0521), indicating that increased NK cell activity in the skin is associated with reduced parasite burden in muscle.

Supplementary Figure 3 Experimental design and NK cell responses in RAG^-/-^ and RAG^-/-^ CD160^-/-^ mice. Schematic overview of the *in vivo* experimental setup comparing RAG^-/-^ and RAG^-/-^ CD160^-/-^ mice (n = 6, n=12 mice per group). Tissues analyzed included skin, skeletal muscle, and blood. Parasite burden was quantified by qPCR in the respective tissues. NK cell activity was assessed by measuring degranulation (CD107a expression) and IFN-γ production.

Supplementary Figure 4 Flow cytometry gating strategy and analysis of NK cells. **(A)** Representative flow cytometry gating strategy used to identify NK cells. Sequential gating included exclusion of debris and doublets, followed by selection of live lymphocytes and NK cell populations. Comparative analysis between RAG^-/-^ and RAG^-/-^ CD160^-/-^ mice is shown, with the double knockout population highlighted in blue.

Supplementary Figure 5 NK cell effector responses in skin and skeletal muscle. **(A)** Total counts of CD107a^+^ NK cells per gram of tissue in skin (left) and skeletal muscle (right), comparing RAG^-/-^ (white circles) and RAG^-/-^ CD160^-/-^ mice (blue circles) under uninfected and *T. cruzi* infected (7 dpi) conditions. **(B)** Total number of IFN-γ NK cells per gram of tissue in skin (left) and skeletal muscle (right), displayed using the same format as in (A). Data are shown on a log10 scale; horizontal lines indicate medians. n = 6 (naive) and n = 12 (infected) per genotype, pooled from two independent experiments. Statistical comparisons across the four groups within each panel were performed using the Kruskal-Wallis test; no significant differences were observed.

Supplementary Figure 6 NK cell maturation states in RAG^-/-^ and RAG^-/-^ CD160^-/-^ mice during *T. cruzi* infection. **(A)** Pie charts showing NK cell maturation states in RAG^-/-^ and RAG^-/-^ CD160^-/-^ mice under uninfected and *T. cruzi* infected conditions. NK cell subsets were defined based on CD11b and CD27 expression. The majority of NK cells displayed a mature CD11b^+^CD27^−^ phenotype across all groups. During infection, RAG^-/-^ CD160^-/-^ mice exhibited an increased proportion of CD11b^+^CD27^−^ NK cells compared to RAG^-/-^ controls (82.3% vs. 69.9%), accompanied by a relative reduction in CD11b^+^CD27^+^ and CD11b^−^CD27^+^ subsets.
