# Supplementary material for: CD160 mediated NK cell activation shapes host defense against acute Trypanosoma cruzi infection in the skin
Source: Front Immunol. 2026 Sep 22;17:1872132. doi: 10.3389/fimmu.2026.1872132 (PMC13638540; doi:10.3389/fimmu.2026.1872132)
Supplement: Supplementary file 1 [file DataSheet1.pdf]

Table 1. Biological Materials and Their Sources

Material	Description
**Human peripheral blood mononuclear cells (PBMCs)**	Isolated from peripheral whole blood of healthy donors at BNITM, Hamburg, Germany. Age: 24–39 years (median: 27). Sex: 10 female, 4 male
**Human dermal fibroblasts (HDFs)**	Isolated from skin biopsies of healthy donors at Elbe Klinikum Buxtehude, Germany. Age: 52–61 years (median: 55). Sex: 3 female, 3 male
**BJ fibroblasts**	ATCC CRL-2522 (foreskin fibroblast cell line). Species: *Homo sapiens*. Age: newborn (<1 year). Sex: male
**C57BL/6J RAG^{-}/$^{-}$ mice**	BNITM, Hamburg, Germany
**RAG^{-}/$^{-}$ CD160^{-}/$^{-}$ mice**	BNITM, Hamburg, Germany
***Trypanosoma cruzi* (Brazil strain)**	Provided by Dr. Maria Fatala Chaben, Instituto Nacional de Parasitología, Buenos Aires, Argentina

Table 2. Buffers and Solutions

Solution	Composition	Source	Use
**FACS buffer**	DPBS + 2% FCS + 2 mM EDTA	BNITM	Flow cytometry
**Binding assay buffer**	DPBS + 5% FCS + 2 mM EDTA	BNITM	Receptor binding assay
**Fixation & Permeabilization buffer**	Foxp3/Transcription Factor Staining Set	Thermo Fisher Scientific (Cat. No. 00-5523-00)	Intracellular staining
**Perm/Wash buffer**	Foxp3/Transcription Factor Staining Set	Thermo Fisher Scientific (Cat. No. 00-5523-00)	Intracellular staining
**MojoSort buffer**	0.5% BSA + 2 mM EDTA in DPBS	BioLegend (Cat. No. TBD)	NK cell isolation

Table 3. Cell Culture Media

Medium	Composition	Use
**BJ medium**	RPMI-1640 supplemented with: 10% (v/v) FCS, 10% Medium 199, 2 mM L-glutamine, 100 U/mL penicillin, 100 µg/mL streptomycin	BJ fibroblast culture
**NK cell medium**	RPMI-1640 supplemented with: 10% human AB serum, 2 mM L-glutamine, 25 mM HEPES, 50 µg/mL gentamicin sulfate	Human NK cell culture

Table 4. Reagents for *In Vitro* Assays

Reagent	Source	Catalog No.	Use
LEGENDplex™ Human CD8/NK Panel (13-plex, V-bottom plate)	BioLegend	741186	Cytokine and cytolytic mediator measurement
MojoSort™ Human NK Cell Isolation Kit	BioLegend	480054	NK cell isolation
RNeasy Mini Kit	Qiagen	74104	RNA isolation
QIAseq Stranded mRNA Select Kit	Qiagen	180445	RNA-seq library preparation
NextSeq 550 System	Illumina	N/A	Bulk RNA sequencing

Table 5. Antibodies for *In Vitro* Assays

Reagent	Clone	Source	Catalog No.	Dilution	Use
hrCD160-His		R&D Systems	6177-CD	25 μg/mL	Recombinant receptor binding
Anti-His-PE	J095G46	BioLegend	362603	1:50	Detection of hrCD160 binding
Anti-HVEM	122	BioLegend	318805	1:50	Detection HVEM
Anti-CD160	688327	R&D Systems	MAB6700	20 μg/mL	NK cell blockade
Anti-CD107a (PE/Cy7)	H4A3	BioLegend	328618	1:100	NK cell degranulation

Table 6. Reagents for mice experiments

Marker	Clone	Catalog No.	Dilution
CD3-BUV395	145-2C11	BD 563565	1:100
CD19-AF700	eBio1D3	BD 557958	1:400
CD45-BUV737	30-F11	BD 748371	1:1000
NK1.1-FITC	PK136	BD 553164	1:50
CD11b-BV650	M1/70	BioLegend 101259	1:400
CD27-BV605	LG.3A10	BioLegend 124249	1:200
CD160-APC	7H1	BioLegend 143011	1:50
CD107a-PerCP-Cy5.5	1D4B	BioLegend 121626	1:50
IFN-γ-PE	XMG1.2	eBioscience 12-7311-82	1:50
